# Bilateral acute angle closure glaucoma as a presentation of isolated microspherophakia in an adult: case report

**DOI:** 10.1186/1471-2415-6-29

**Published:** 2006-07-07

**Authors:** Sushmita Kaushik, Nishant Sachdev, Surinder Singh Pandav, Amod Gupta, Jagat Ram

**Affiliations:** 1 Department of Ophthalmology, Postgraduate Institute of Medical Education and Research, Chandigarh, India

## Abstract

**Background:**

Bilateral simultaneous angle closure glaucoma is a rare entity. To our knowledge this is the first reported case of bilateral acute angle-closure glaucoma secondary to isolated microspherophakia in an adult.

**Case presentation:**

A 45-year-old woman presented with bilateral acute angle closure glaucoma, with a patent iridotomy in one eye. Prolonged miotic use prior to presentation had worsened the pupillary block. The diagnosis was not initially suspected, and the patient was subjected to pars-plana lensectomy and anterior vitrectomy for a presumed ciliary block glaucoma. The small spherical lens was detected intraoperatively, and spherophakia was diagnosed in retrospect. She had no systemic features of any of the known conditions associated with spherophakia. Pars-plana lensectomy both eyes controlled the intraocular pressure successfully.

**Conclusion:**

This case demonstrates the importance of considering the diagnosis of isolated microspherophakia in any case of bilateral acute angle closure glaucoma. Lensectomy appears to be an effective first-line strategy for managing these patients.

## Background

Bilateral simultaneous acute angle closure is a rare entity, infrequently reported after psychotropic drug intake, [1-3] general anesthesia, [4] or snake bite [5]. Spherophakia is an uncommon condition in which the small, spherical lens may led to pupillary block and secondary angle closure glaucoma. We present a case of isolated microspherophakia presenting as bilateral acute angle-closure glaucoma in a middle-aged woman.

## Case report

**Figure 1:**
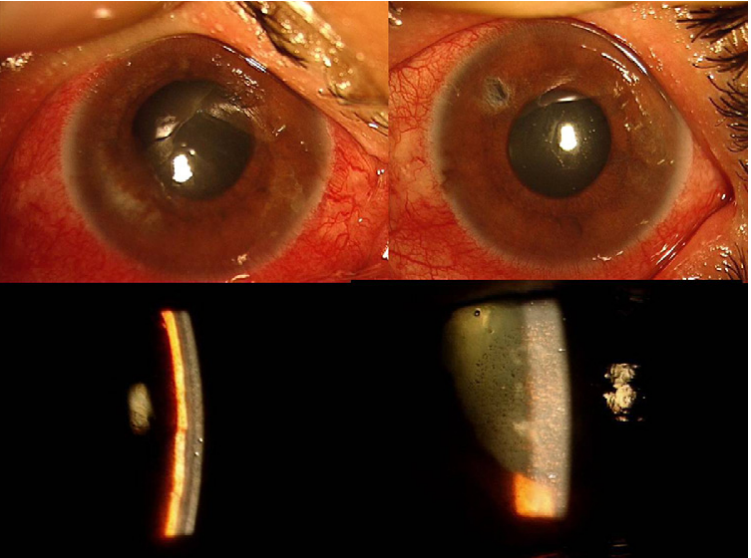
(Top) Slit-lamp photograph of the both eyes at presentation showing circumciliary congestion and corneal haze. Note the iridotomy in the left eye. (Bottom left) Slit section showing flat anterior chamber with iris apposed to posterior corneal surface. (Bottom right). Diffuse pigmentation seen at the posterior corneal surface.

**Figure 2:**
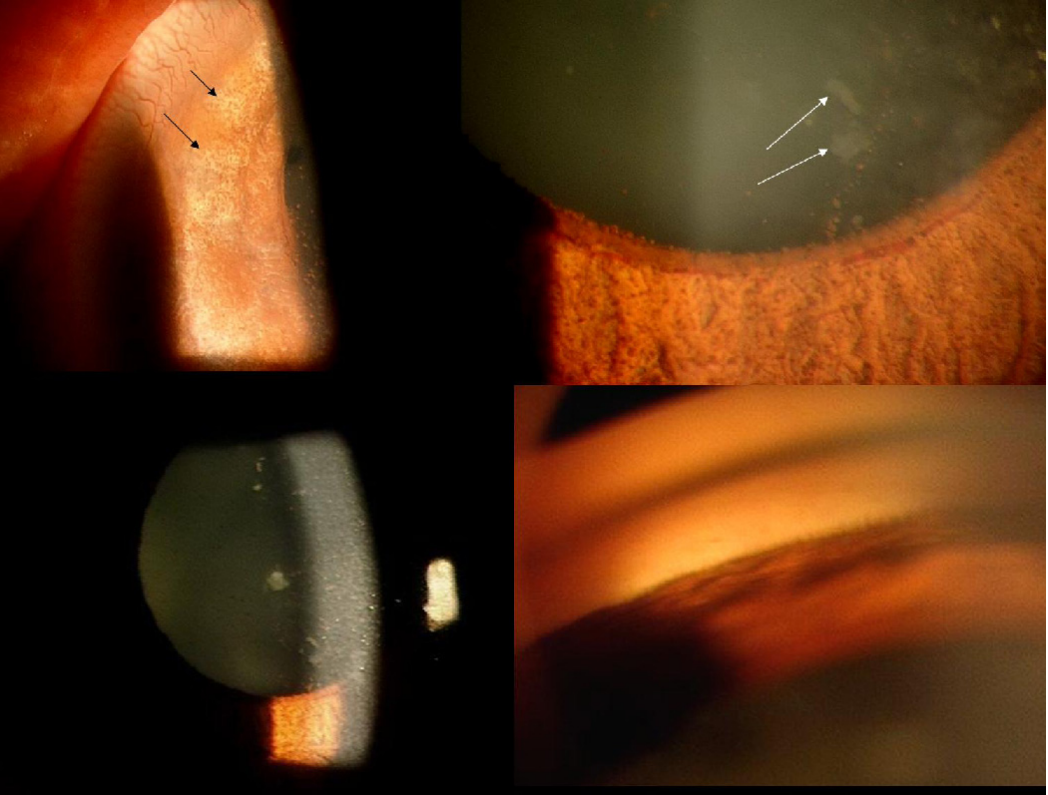
Magnified picture showing signs of acute angle closure: patches of iris atrophy. (Top left), dilated iris vessels (Top right), and glaucomflecken (Top right and Bottom left). (Bottom right) Completely closed angles on gonioscopy

**Figure 3:**
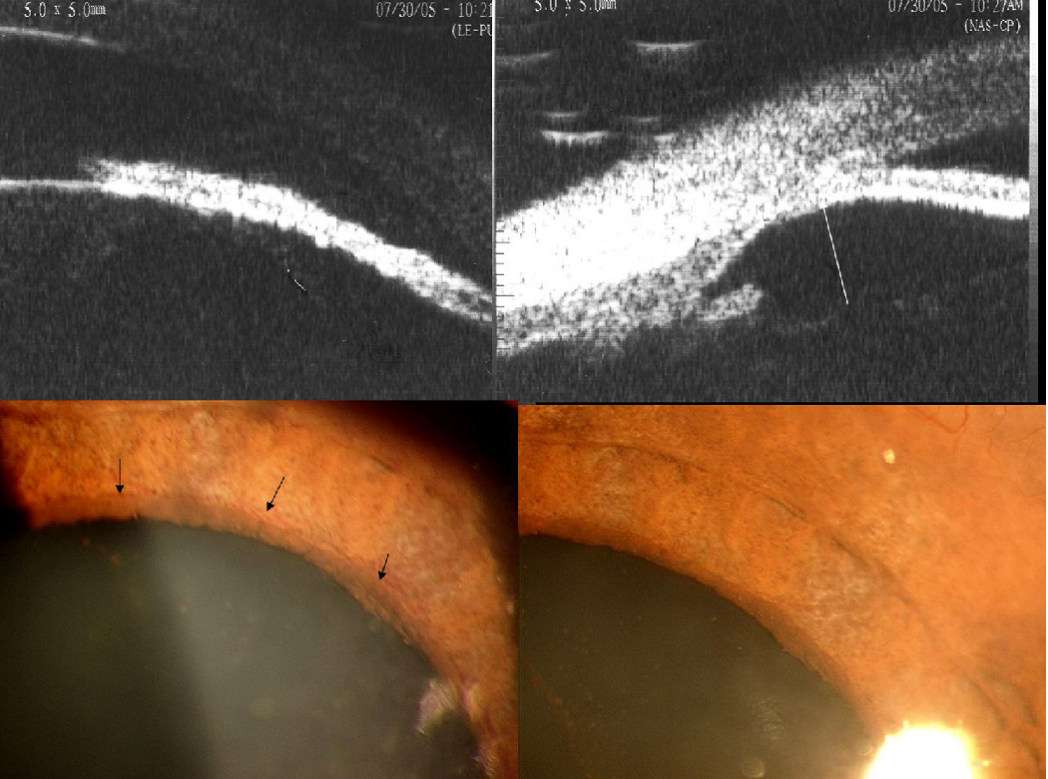
(Top left) Ultrasound Biomicroscopic scan of the right eye showing anteriorly displaced crystalline lens and forward movement of entire iris-lens diaphragm. (Top right) UBM scan of the left eye showing obliteration of the peripheral anterior chamber by extensive synechiae. (Bottom left) Prominent iris vessels at presentation, which regressed (bottom right) after control of IOP.

A 45-year-old Nepalese woman presented with acute pain and decreased vision in both eyes since two months. She had a history of treatment with oral acetazolamide, 4% pilocarpine drops and underwent laser iridotomy in the left eye. There was no previous history of such episodes. At presentation (Fig. 1, Top), both eyes had light perception vision, intraocular pressure (IOP) was 50 and 54 mmHg in the right and left eye respectively. The left eye had a mid-peripheral patent laser iridotomy (Figure 1, top right). The anterior chambers were nearly flat (Figure 1, bottom left), with diffuse pigmentation on the posterior corneal and anterior lens surface (Figure 1, bottom right). Both eyes showed signs of acute angle closure (Figure 2, top), with iris atrophy, glaucomflecken and prominent iris vessels (Figure 2, top right) and closed angles on indentation gonioscopy (Figure 2, bottom right). Ultrasound Biomicroscopy (UBM) showed an anteriorly displaced crystalline lens with extensive irido-lenticular contact and peripheral anterior synechiae (PAS) closing the angles completely in both eyes (Figure 3, top). Retinoscopy was tried, but was not possible owing to the poor fundal glow due to diffuse pigmentation on the posterior corneal surface. The axial length was 21.63 mm and 22.52 mm in the right and left eye respectively. B-Scan ultrasonography showed normal posterior segments in both eyes.

The patient was given Injection Mannitol 20% 350 ml stat. followed by systemic acetazolamide 250 mg four-times-a day, Syrup Glycerol 30 ml thrice-a-day, topical timolol maleate 0.5% twice-a-day and Brimonidine 0.15% twice-a-day. Pilocarpine was withheld since the iris-lens diaphragm was anteriorly displaced. The IOP reduced to 34 and 38 mmHg respectively.

**Figure 4:**
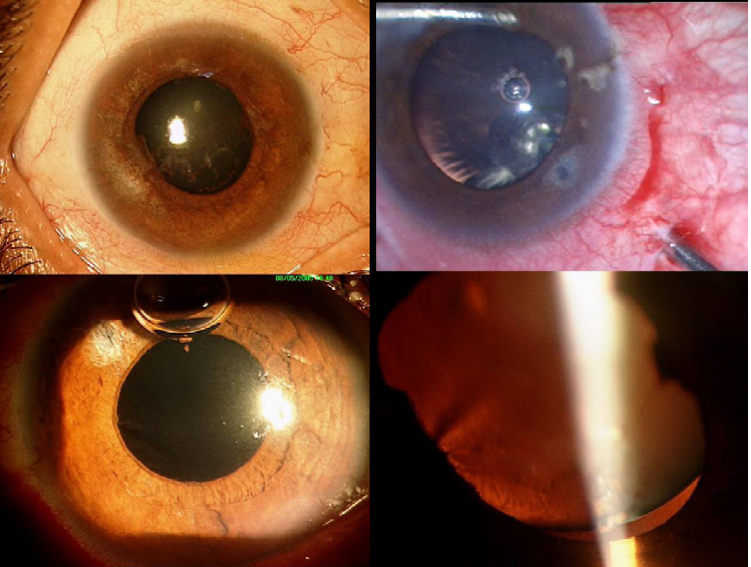
(Top left) Slit-lamp photograph of the right eye following cycloplegic therapy showing relief of the acute angle closure. Note the decreased ocular congestion and clear cornea. (Top right) Intra-operative photograph of the left eye showing the edge of the crystalline lens within the pupillary border. (Bottom left) First post-operative day of the left eye after undergoing a pars-plana lensectomy and anterior vitrectomy. (Bottom right) Picture of the right eye after dilatation with phenylephrine showing clearly the small and spherical crystalline lens with the lens edge visible within the pupillary border.

There was no history suggestive of any of the reported causes of bilateral acute angle closure such as psychotropic drug intake [1-3] general anesthesia [4] or snake bite [5]. A possibility of ciliary block glaucoma owing to prolonged unrelieved angle closure was kept in mind [6,7]. Atropine sulphate1% drops were added thrice a day, following which the acute congestive phase was relieved with regression of the prominent iris vessels seen during the acute phase (Figure 3, bottom). Following atropine treatment, the IOP further reduced to 30 and 34 mm Hg and the patient was symptomatically better (Figure 4, top left).

This response to cycloplegic treatment strengthened the possibility of a ciliary block glaucoma, and the patient underwent a pars-plana lensectomy and anterior vitrectomy (PPL-AV) in the left eye. Intraoperatively, under pupillary dilatation, the lens was found to be small and spherical (Fig 4, top right), suggestive of microspherophakia. Following surgery (Fig 4, bottom left), the IOP reduced to 12 mm Hg without medication with total relief of symptoms.

The right eye was re-examined under dilatation. The lens was small and spherical with the lens edge seen within the pupillary margin (Fig 4, bottom right). Microspherophakia was diagnosed in retrospect, with prolonged inverse angle closure glaucoma. We assessed the patient with particular reference to systemic conditions associated with spherophakia, such as Weill-Marchesani's syndrome, Marfan's syndrome and homocystinemia. She was of average height (155 cm), and moderately built. She had normal skeletal proportions with no evidence of arachnodactyly, short and stubby fingers or reduced joint mobility. There was no anterior chest deformity or scoliosis. The cardiovascular examination was within normal limits. Urine chromatography for homocystinuria was negative.

**Figure 5:**
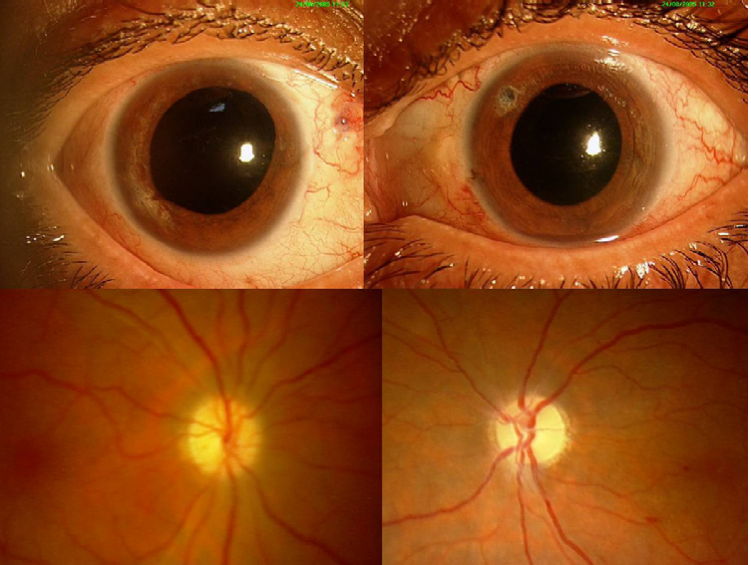
(Top) Final picture of both eyes after surgery. (Bottom left) Pale neuroretinal rim (NRR) of the right eye following prolonged optic nerve head ischemia. (Bottom right) Optic nerve head of the left eye showing advanced glaucomatous optic neuropathy, and pale NRR.

**Figure 6:**
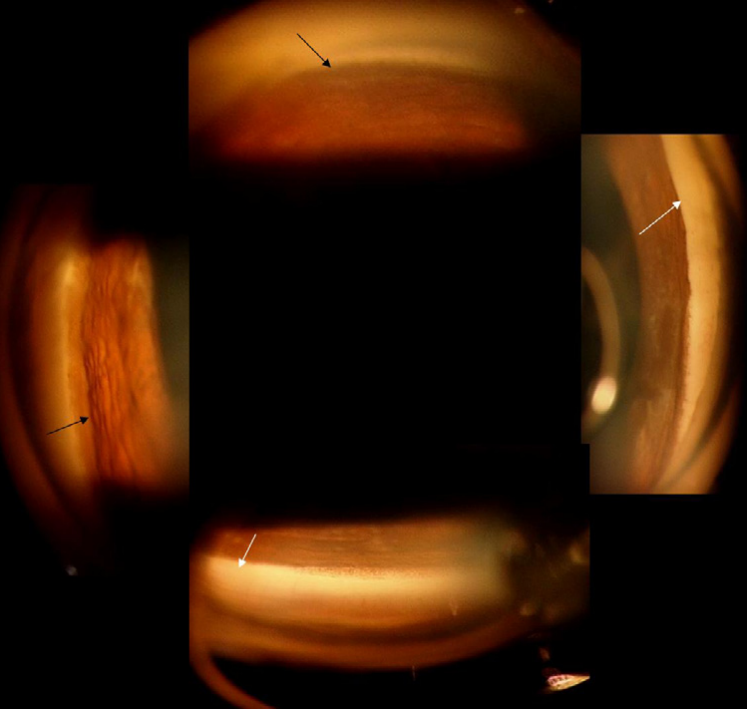
Post-operative gonioscopy pictures of the right eye showing partially opened angles (black arrows) especially in inferior and nasal angles, and also areas of synechial closure (white arrows) in the superior and temporal angle.

The patient underwent PPL-AV in the right eye. At last follow-up six weeks later, the IOP in both eyes remained controlled without anti-glaucoma medication (Figure 5, top), with pale optic discs secondary to prolonged ischemia (Figure 5, bottom). Post-operative gonioscopy showed the angles to have partially opened in both eyes (Figure 6). The best-corrected-visual-acuity was counting fingers close to face in both eyes with refractive correction of +11. and +11.0 Diopters in the right and left eye respectively.

Despite our best efforts, the patient could not be contacted in rural Nepal for subsequent follow-ups. This also resulted in our inability to obtain informed consent from the patient for publication of this case report.

## Discussion

This is the first reported case of microspherophakia presenting as bilateral simultaneous acute angle closure glaucoma in an adult. The condition has been reported in a child, [8] where the underlying cause was unsuspected, and Pilocarpine aggravated the pupillary block, as was probably the situation in our patient. Microspherophakia is usually associated with systemic disorders such as Weill-Marchesani's' syndrome, homocystinemia, Marfan's syndrome, Alport's syndrome and Klinefelter's syndrome [9-12]. Our patient had no features suggestive of any of these conditions. Glaucoma in isolated microspherophakia is less commonly described [11,12]. It can result from several mechanisms: pupillary block by the spherical lens, irritation of the ciliary body by the dislocated lens [13], or by complete luxation of the lens in anterior chamber. Unrelieved pupillary block may lead to peripheral anterior synechiae (PAS) formation and irreversible trabecular damage. Chronic pupillary block without complete angle closure may lead to crowding of the trabeculae by the spherophakic lens [11].

Our patient presented with bilateral acute angle closure secondary to pupillary block, which was worsened by miotics and relieved to some extent by cycloplegic treatment. Urbanek [14] described this phenomenon as inverse glaucoma. We did not suspect spherophakia initially, given the age group (most patients present in adolescence or early adulthood), [8,15-17] and her presentation as bilateral acute angle closure glaucoma with dilated iris vessels simulating iris neovascularization. The anteriorly displaced lens-iris diaphragm and the extensive irido-lenticular contact seen on the UBM prompted a consideration of malignant glaucoma following prolonged angle closure. Malignant glaucoma has been described without a history of laser or surgery [18,19], and after prolonged miotic use for angle closure glaucoma [6,7]. The partial resolution of her condition seen after atropine treatment for the presumed ciliary block further consolidated our suspicion. Inverse glaucoma would respond in an identical manner, which in fact was what happened in our patient. Spherophakia was diagnosed only in retrospect, once we visualized the lens edge within the dilated pupil during the lensectomy procedure.

The management of glaucoma in spherophakia is still debated. Willoughby et al [20] described a case of spherophakia with glaucoma whose IOP could be successfully controlled without additional medication following lensectomy. In contrast, Yasar [17] described a patient in whom lensectomy could control the IOP in the short-term, but who subsequently required mitomycin-C augmented trabeculectomy in both eyes. Kanamori et al [15] reported good IOP control with goniosynechiolysis and lensectomy in a patient of spherophakia and chronic angle closure glaucoma. Asaoka et al [21] reported trabeculectomy to control the IOP in a patient with spherophakia, but open angles.

The IOP in our patient remained controlled without medication for the six weeks that we could follow her up, before she went back and never returned. The angles did appear to have opened partially, but it must be kept in mind that although lensectomy will relieve a pupillary block, it may not suffice to control the IOP in case of the presence of extensive PAS. Only longer follow-up can indicate how effective this procedure would be for our patient.

## Conclusion

It is important to include spherophakia in the differential diagnosis of bilateral narrow angle glaucoma in adults, and remember that prolonged miotic therapy may lead to worsening of the condition. Pars-plana lensectomy appears to be a reasonable first-line treatment strategy for the glaucoma. The possibility of uncontrolled IOP despite lensectomy must be kept in mind, especially in the presence of extensive peripheral anterior synechiae.

## Abbreviations

1. IOP – Intraocular Pressure

2. PAS – Peripheral Anterior Synechiae

3. UBM – Ultrasound Biomicroscopy

4. PPL-AV – Pars-plana lensectomy and Anterior Vitrectomy

## Competing interest statement

The author(s) declare that they have no competing interests.

## Authors' contributions

SK diagnosed, managed the case, and wrote the final paper, NS drafted the manuscript, SSP and AG gave valuable suggestions for the management particularly regarding the decision of vitrectomy, and JR critically reviewed the manuscript. All authors read and approved the final manuscript.

## Pre-publication history

The pre-publication history for this paper can be accessed here:


